# Experimental Investigation on the In-Plane Creep Behavior of a Carbon-Fiber Sheet Molding Compound at Elevated Temperature at Different Stress States

**DOI:** 10.3390/ma13112545

**Published:** 2020-06-03

**Authors:** David Finck, Christian Seidel, Anika Ostermeier, Joachim Hausmann, Thomas Rief

**Affiliations:** 1 SIEMENS AG, Corporate Technology REE MDM POL-DE, 91058 Erlangen, Germany; christian.seidel@siemens.com (C.S.); anika.ostermeier.ext@siemens.com (A.O.); 2 IVW—Institute for Composite Materials GmbH, Component Development, 67663 Kaiserslautern, Germany; joachim.hausmann@ivw.uni-kl.de (J.H.); thomas.rief@ivw.uni-kl.de (T.R.)

**Keywords:** creep, relaxation, composites, sheet molding compound, SMC

## Abstract

The creepage behavior of one thermosetting carbon fiber sheet molding compound (SMC) material was studied applying in-plane loading at 120 °C. Loads were applied in bending, tension and compression test setups at the same in-plane stress level of 47 MPa. Different creep strain rates were determined. The creep strain rate in flexural loading was significantly higher than in tensile loading. The test specimens in compression loading collapsed within minutes and no findings regarding the creep strain rates were possible. Overall, it was observed that the thermosetting press resin of this industrially used material had only little creep load bearing capacity at the mentioned temperature when loaded in mixed stress states. The test data has high usage for estimating design limits of structural loaded SMC components at elevated temperature.

## 1. Introduction

Creep is the irreversible increase in deformation of a material under a load [1]. When developing components with polymers, it is important to be aware of the creep behavior of the material used. Creep can lead to dramatic component failure; significantly lower strength than the static mechanical ones are possible. The steadily increasing deformation can also lead to failure, for example, if tolerance levels are exceeded. The problem with fiber composites here is their complex creep behavior, which cannot easily be estimated or simulated.

In this paper, the tensile, compressive, and flexural creep strain rates of an industrially used carbon fiber sheet molding compound (SMC) at a temperature of 120 °C and a stress of 47 MPa are compared. There is no known publication in which these investigations were carried out in a comparable way for a SMC. Creep experiments were conducted in this study by exposing test specimens at different stress states in an oven at 120 °C to a constant external load and tracking the strain over an extended timespan. The temperature and the stress in the test specimen were held constant during the experiment.

The creep of composite materials depends mainly on the creep behavior of the matrix [2,3]. Since the matrix is stressed differently under varying load conditions, different creep strains are also to be expected. For SMCs, the matrix is always required for the mechanical force transmission between the fiber rovings. A noticeable creep behavior was, therefore, expected at every stress state for the SMC.

Overall, the operational capability of the presented SMC at this temperature was quite limited, as the creep test results demonstrated. These results were unexpected as the test temperature was still clearly below the glass transition temperature of the material [4,5].

A related study was conducted for creep strains in the thickness direction at 120 °C, [6]. As mentioned there, 120 °C was selected as this is an often-mentioned upper temperature limit for structurally used polymers in the mobility sector [2].

## 2. Literature Research

### 2.1. The Creep Mechanism

Creep mechanically occurs at atomic level and is caused by thermally activated diffusion processes. The creep progress is typically divided into three stages. In the first stage, the material is exposed to a high creep strain rate, which weakens considerably in the transition to the second stage. In the second stage, the creep strain rate remains at an approximately constant level. In the third stage, an accelerated creep strain occurs again as cracks and flaws form in the material. These lead to locally strong increased stresses until the specimen finally collapses [1,5,7,8,9,10].

There are publications that deal with the mechanical processes during the different creep stages in detail [5,9,10]. In particular, [10] deals with the creep mechanics of non-metallic materials.

### 2.2. Anisotropic Material Behavior of Random-Oriented Fiber-Reinforced Composite Materials

The literature suggests that randomly oriented fiber-reinforced composites can be approximately computationally considered using a quasi-isotropic endless reinforced laminate [2]. However, many production processes of randomly oriented composites generate a certain preferential fiber orientation in the material. In these cases, the application of a quasi-isotropic laminate seems inapplicable. This preferential fiber orientation for a compression molded test plate was investigated in a publication in detail using computer tomographic imaging [11]. Another publication deals directly with the anisotropic creep behavior of randomly oriented natural fiber composites processed in a continuous extrusion process [12]. Both studies exhibited significant preferential fiber orientations in the investigated composites and, therefore, mechanical properties that vary with different preparation angles from the test plates.

**Figure 1:**
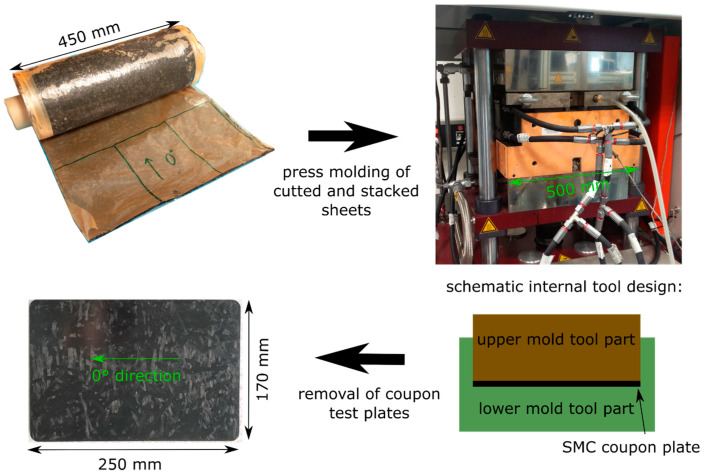
Processing of carbon fiber SMC ‘SMCarbon 80 CF60-3K/2’ from manufacturer Polynt into coupon test plates.

In the following investigations, the in-plane anisotropy of the SMC material with respect to the creep effect is not discussed in detail. However, to ensure comparability between the different load cases, the coupon and load directions were kept the same for all investigations. Therefore, the unwinding orientation of the SMC roll is set to 0° orientation (see Figure 1). The SMC sheets were cut in the 0° direction and pressed in a homogenous stack-up. All investigated test specimens were prepared in the 0° direction.

### 2.3. Influence of Different Stress States on Composite Creep Behavior

Similar studies examined creep at different stress states on a composite material [13,14]. In both studies, creep compliance curves were presented for endless fiber composites. The creep compliance can be understood as the summed-up creep strain per unit of stress [15].

Goertzen et al. compared tensile and flexural loadings on a carbon fiber epoxy composite [14]. The tested stresses were not the same in both loadings. A comparison of tensile and flexural creep compliances indicated that higher creep strains were obtained in flexural loading. Nevertheless, the authors assumed that no nonlinear material effects occurred at different test stresses.

A NASA study by Gates et al. investigated the creep behavior at tensile and compression loading [13]. A thermoplastic polyamide K3B matrix with IM7 carbon fibers was studied. The creep compliance curves presented a mixed impression. When the creep tests started, the creep strains were higher at tensile loading. The creep strains in compression loading crossed the tensile ones after several hours and raised higher afterwards.

## 3. Material and Processing

One carbon fiber SMC from the manufacturer, Polynt (Miehlen. Germany), declared as Polynt SMCarbon 80 CF60-3K/2, was examined in this study. The SMC was made with an epoxy-based matrix with the use of a 3 K roving, and cut into lengths of 25 mm to 50 mm. Fiber weight fraction was stated as 60% in the datasheet. All produced test coupons were tempered for 2 h at 160 °C prior to testing [16].

The SMC was processed in a compression molding tool that can produce 170 mm wide × 250 mm long test coupons (see Figure 1). Typical in-mold pressure was 100 bar during the processing. The covered mold area was around 80%. This value was comparably high to typical covered mold areas in production of SMC parts. The high proportion of the covered mold area resulted in relatively low material flow effects. The thickness of the produced SMC coupons was h = 3.65 mm. All test specimens were prepared with a diamond studded carbon fiber reinforced polymer (CFRP) saw blade on a table saw.

**Figure 2:**
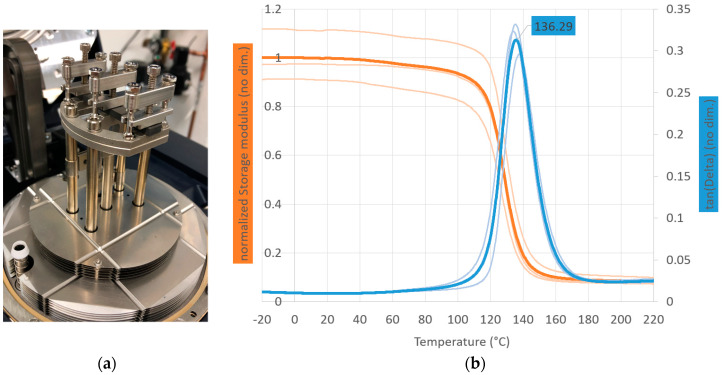
Dynamic mechanical analysis (DMA) in a DMA 850 (TA Instruments Inc., New Castle, PA, USA) of SMC material Polynt SMCarbon 80 CF60-3K/2: (**a**) Dual-cantilever measuring setup for determining the glass transition; (**b**) storage-modulus- and tan(δ)-graph from −20 to 220 °C.

The glass transition temperature was measured in a dynamic mechanical analysis (DMA) in a dual-cantilever setup (tan-Delta-method, 0.005% strain, 1 Hz, 3 K/min) to 136 °C using the mean of three measurements (Figure 2). The dual-cantilever setup, which is shown in Figure 2, is a common test setup for fiber reinforced polymers in DMA, since relatively high stresses can be built up with small forces. The specimen in the dual-cantilever setup is subjected to a mixture of bending and shearing. An advantage over a pure bending design is that the specimen is clamped at all three support points. Therefore, no disturbance of the measurement due to uneven sample support is to be expected, as it could occur with a pure bending setup. A disadvantage is that the sample stress does not allow a direct comparison with the expected thermomechanics in the bending, tensile, or compression test. Nevertheless, the test provides an estimate of the decrease in mechanical properties with increasing temperature and allows a reliable prediction of the glass transition of the matrix material [17].

Static mechanical flexural testing was conducted at an ambient temperature and 120 °C with 10 specimens per test. Test results are summarized in Table 1.

The flexural strength values were used to find an appropriate stress level for further creep testing. The flexural strength at 20 °C was in good accordance with the value of 520 MPa stated in the datasheet [16].

## 4. Experimental Results

### 4.1. Creep under Flexural Load

**Figure 3:**
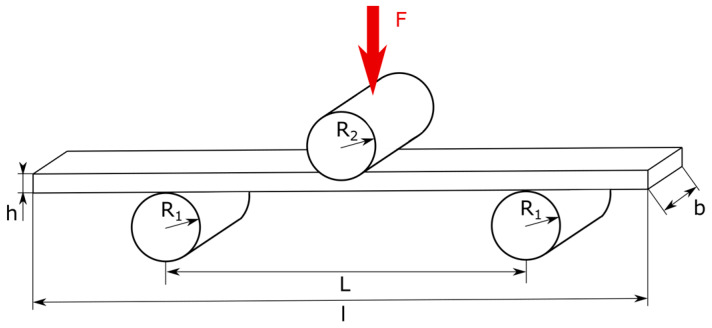
Three-point flexural test setup according to DIN EN ISO 14125 [19].

The creep behavior under flexural load was investigated in a custom-made test fixture. The test fixture was built according to DIN EN ISO 14125 as a three-point bending setup (Figure 3) [19]. As declared in the stated standard, the specimen dimensions were b = 15 mm wide × l = 80 mm length. The radii of the support rods were R_1_ = R_2_ = 5 mm. L = 64 mm was the distance between the lower support rods.

**Figure 4:**
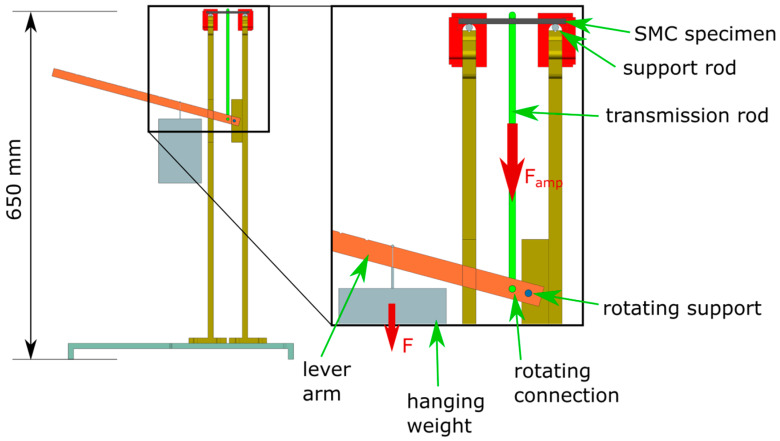
Schematic drawing of the used test fixture for measurement of creep deformation under flexural load.

The test load on the specimens was applied by a hanging weight, where weight force was multiplied by a lever arm construction (Figure 4). The test fixture was designed for the realization of different stresses, which is why the weights could be hung in notches at discrete positions along the lever arm.

**Figure 5:**
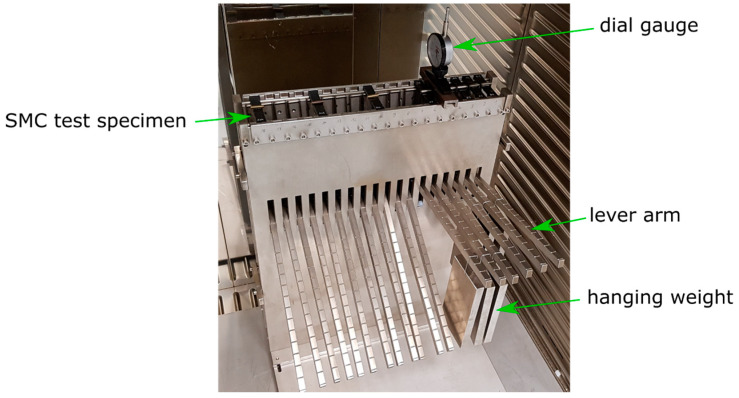
Deformation measurement with a dial gauge on the used flexural test fixture.

The test fixture was stored in an oven at 120 °C for the tests. The gradual deformation of the bending specimens was measured with a dial gauge at discrete times (see Figure 5). The dial gauge had a scale resolution of 0.01 mm. A gauge repeatability study was conducted to estimate the misreading at measurements. The relative error was around 0.00034% (1-sigma) for 20 measurements. The misreading error was added to the error bars of flexural testing in Figure 9.

The stress during the test was kept constant by the 1.5 kg hanging weight, which exerted the same weight force regardless of the deformation of the specimen. The hanging weight applied the stress on the specimen immediately. Due to the number of weights, around one minute passed until the first measurement was taken. The experiment was conducted for 300,000 s (83.3 h). The in-plane stress in the specimens at this test setup was calculated to 47 MPa by Equation (1) [19].

The amplified weight was measured with a hook scale to 10.0 kg, which could be calculated to a weight force of $F_{amp}$ = 98.1 N.

Due to the notches on the lever arm, which were attached at fixed positions, only certain stress values could be realized. The stress value of 47 MPa was set constant for the following tests. Therefore, a comparison of the different load cases was possible.

(1) $$\sigma =\frac{3\times F_{amp}\times L}{2\times b\times h^{2}}$$

The creep strain was calculated from the measured deformation (s) and the cross-section of the specimen (see Equation (2)) and is shown in Figure 9 [19]. The elastic strain component, due to the weight already hanging at the beginning of the test, was not included in the creep strain.

(2) $$\varepsilon_{creep\_flexural}=\frac{6\times s\times h}{L^{2}}$$

### 4.2. Creep under Tensile Load

**Figure 6:**
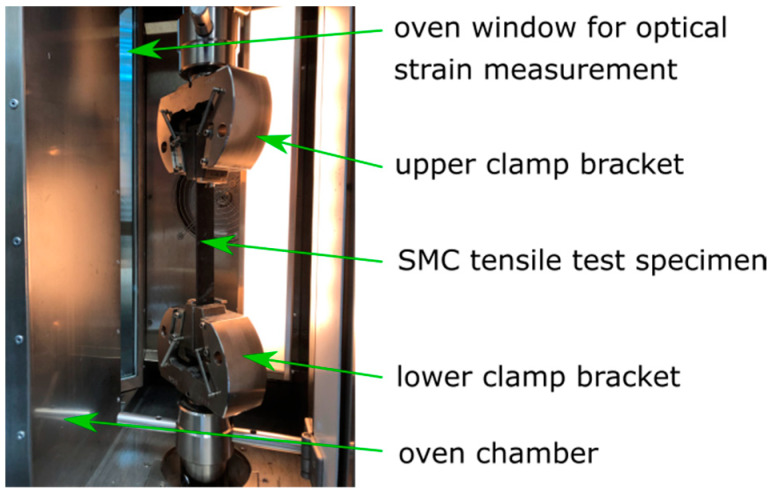
Tensile creep test in an oven chamber of a universal testing machine.

The creep behavior in tensile direction was investigated in the tensile fixture of a 50 kN universal testing machine. The test was conducted according to DIN EN ISO 527-4 [20]. The test specimens were measured here as b = 25 mm wide × l = 250 mm long. The universal testing machine was programmed to apply a specified force within around one minute and then readjust the applied force to a constant value while testing. Around the test setup was an oven chamber, which was heated to 120 °C before the test started (see Figure 6). The gradual deformation of the tensile specimens was observed through a furnace window with an optical camera measuring system.

The optical measurement system videoXtens (ZwickRoell GmbH & Co. KG, Ulm, Germany) with a resolution of 0.001 mm was used here [21]. The system measured the distance between two measuring marks optically in the center of the specimen and calculated the strain of the specimen. Based on the difference in contrast between the white and black surface, the software could determine the position of the measuring mark. The measuring marks were spaced 50 mm initially. The adhesive measuring marks were applied manually. The test time was 300,000 s (83.3 h) and every 10 s, a measurement was taken. In the evaluation (see Figure 9), however, only every 10,000 s measured values are shown.

### 4.3. Creep under Compression Load

**Figure 7:**
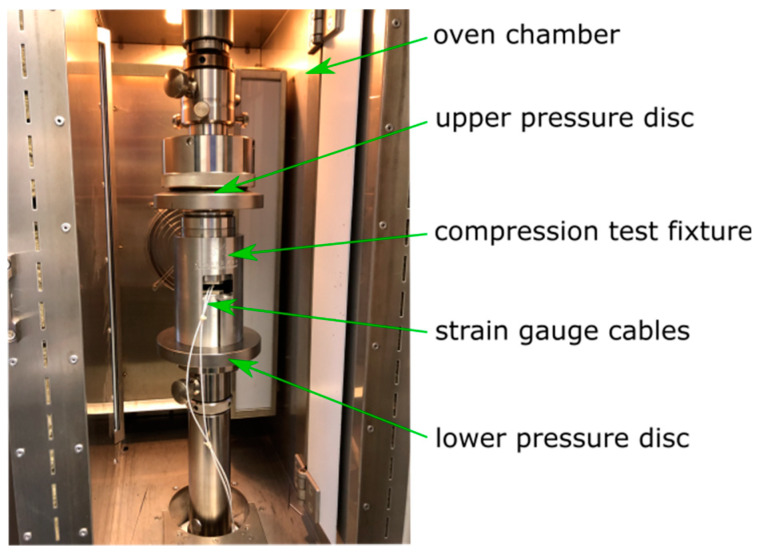
Compression creep test fixture in an oven chamber of a universal testing machine.

The creep behavior of the SMC was also investigated in a compression fixture of a 50 kN universal testing machine. The test was conducted according to DIN EN ISO 14126 [22]. The universal testing machine was programmed as described for tensile load. The test specimens were measured here as b = 10 mm wide × l = 110 mm long. The unsupported test area was 10 mm in length and 50 mm of each side of the specimen were clamped. Sample observation, via the optical measuring system, was not possible here because the specimen was surrounded by the compression test fixture (see Figure 7).

**Figure 8:**
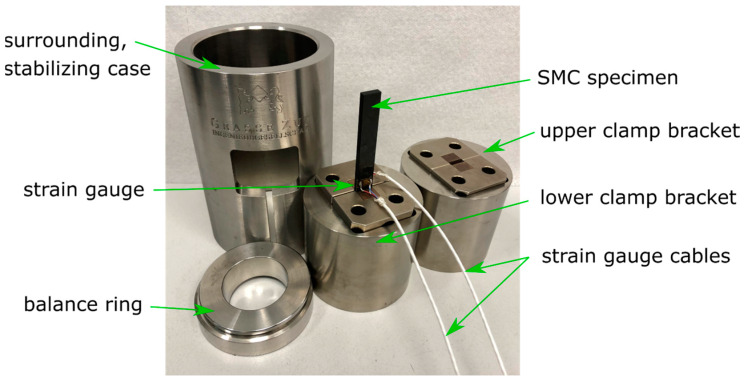
Compression creep test fixture build apart.

Instead, the gradual deformation of the specimens was measured by two strain gauges applied to the specimen (see Figure 8). The strain gauges were applied with a high temperature adhesive to minimize disturbing creep effects from the bonding.

A balance ring with a ball joint on top of the fixture ensures an even load distribution.

When applying the strain gauges, care was taken to ensure that the alignment was vertical on the specimen. The strain gauges were applied to an adhesive tape and then aligned with marks on the tape.

The gradual deformation of the compression specimens was regularly determined by measuring the resistance of the strain gauges. The strain gauge measurement system QuantumX (Hottinger Baldwin Messtechnik GmbH, Darmstadt, Germany) with a resolution of 1 µm/m or 0.0001% strain was used for this purpose [23]. One strain gauge had two terminals. Two wires were connected with every terminal. Therefore, it was possible to measure the resistance of the electric wiring separately from the strain gauge. By this procedure, the electric wiring resistance could be excluded by the strain measurement software. No temperature compensation was needed for the strain gauges, as the signal was zeroed at the beginning of measurement and the temperature remained constant afterwards. Strain gauges were applied on both sides of the specimen for recognition of uneven strain distribution or even buckling of the test coupon. The mean strain value of both strain gauges was evaluated in the software.

The test fixture was stored in an oven at 120 °C for the tests.

## 5. Comparison of Creep Strain over Time at Different Stress States

**Figure 9:**
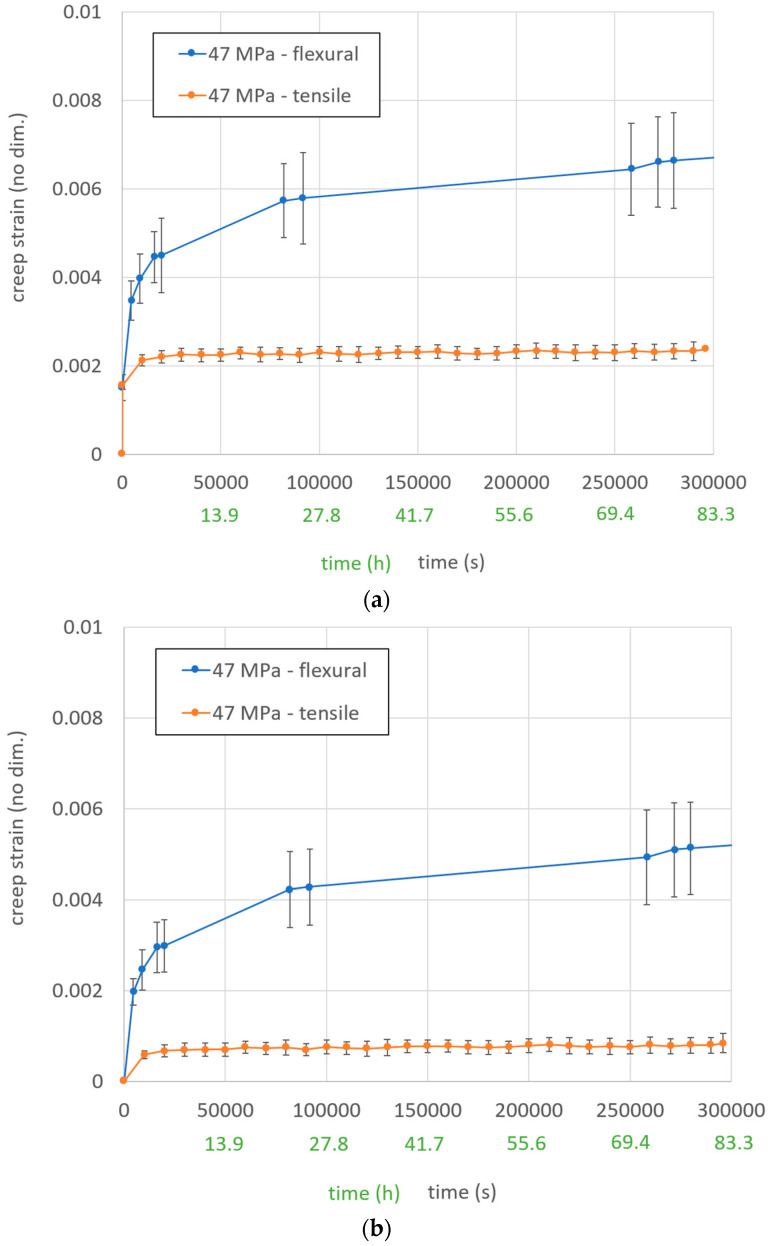
Comparison of different stress states at the same stress level for: (**a**) total strain over time; (**b**) creep strain over time.

**Figure 10:**
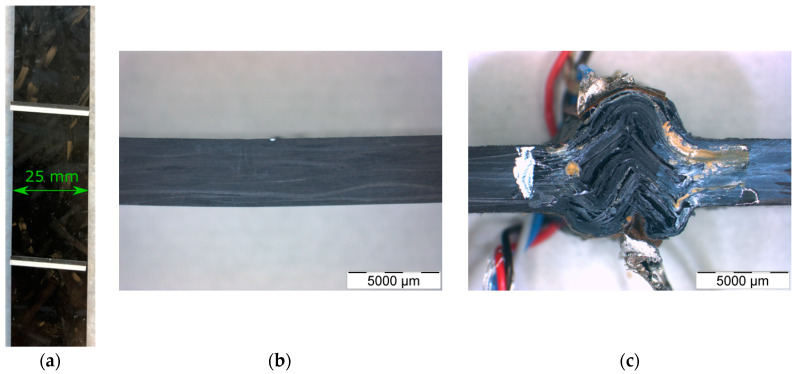
Tested creep specimens of Polynt SMCarbon 80 CF60-3K/2 at 47 MPa in-plane stress. (**a**) after 84 h in a tensile test setup; (**b**) after 84 h in a bending test setup; (**c**) collapsed after 5 min in a compression test setup.

Different strain measurement graphs over time are presented in Figure 9. The graphs display the mean tensile and flexural strain over time out of three measurements and the standard deviation with vertical bars. The total strain is a combination of the elastic and creep displacement. The creep strain graph does not include initial elastic displacements by the applied loads. The compression creep test results are not shown in Figure 9 as all three specimens completely collapsed after 5–85 min (see Figure 10). The test signals of the strain gauges were lost at this point.

In retrospect, it would have been more reasonable to select a stress for the tests where creep test specimens did not buckle. However, the specimen behavior showed illustratively how significant the mechanical differences were for different types of loading (see Figure 10).

The graphs of creep strain were in good agreement with expected graphs from the literature [1]. The primary and secondary creep stages were clearly recognizable. There was a significant deviation between tensile and flexural in-plane creep strain over time at the same in-plane stress level. The creep strength of the material was extraordinarily lower in compression than in tensile loading. Therefore, it seems to be reasonable that the flexural creep strains were significantly higher than the tensile creep strains, since the stress includes tension and compression.

Figure 10 shows representative specimens from Section 4. The tensile specimen revealed no externally visible alteration. The bending test specimen had a small permanent deflection of roughly 0.7 mm. The compression test specimen was destroyed in the testing area and no longer had any residual load-bearing capacity. It was easy to see that buckling of the specimen had occurred. The buckling of the specimen during the creep test was probably due to the programming of the universal testing machine, which ensured that the force was always kept constant. This meant that if the specimen failed, it would continue to press.

## 6. Conclusions and Outlook

The conducted experiments revealed that:

- Different stress states result in significantly different creep strain rates for the investigated SMC on the same stress value. Since the matrix experiences different load carrying shares at the individual load cases, the various strain rates had been expected.
- The creep strains in flexural loading were significantly higher than in tensile loading.
- It was observed that the creep strength for the mentioned material was significantly lower under compression than under tensile or bending. Therefore, it was assumed that the higher creep strains under flexural loading, which was a mixed stress loading, were caused by the compression loading shares.
- Literature research on expectable creep behavior of endless fiber composites was already indicating that the creep strains under flexural loading would be higher than under tensile loading [14]. As creep strains under tensile and compression loading could not be compared here, no comparison could be made with a study by NASA [13].
- It was also shown that the presented thermosetting press molding material, with a relatively high glass transition temperature of 136 °C reinforced with chopped fibers, could suffer from creep at a temperature of 120 °C. As this temperature is still in range for different applications, such as the automotive application, the creep behavior of these materials has to be considered for the development of structural loaded components [2].
  The automotive industry was selected because an increasing interest in SMC sector was found in different publications and developments [24,25,26].

## Figures and Tables

**Table 1:** Measured mechanical material properties of investigated carbon fiber SMC [18].

Material Property	Mean Value ± 1 SD	Test Standard
Flexural strength in-plane at 20 °C	589 MPa ± 64 MPa	DIN EN ISO 14125 [19]
Flexural strength in-plane at 120 °C	373 MPa ± 21 MPa	DIN EN ISO 14125 [19]
